# Safety and Readmission in Pediatric Ambulatory Surgery in a Tertiary Hospital

**DOI:** 10.7759/cureus.21274

**Published:** 2022-01-15

**Authors:** Assem M Alghamdi, Saud A Aljadaan, Saif A Alsemairi, Moath A Alowairdhi, Mohammed A Alhussain, Rumyyan A Alrumyyan

**Affiliations:** 1 College of Medicine, King Saud Bin Abdulaziz University for Health Sciences, Riyadh, SAU; 2 Pediatric Surgery, King Abdulaziz Medical City, Riyadh, SAU

**Keywords:** ambulatory surgery, complications, outcomes, pediatrics, readmission, safety

## Abstract

Objectives: To assess the safety and outcome of pediatric ambulatory surgery by measuring the rate of complications and readmission and identifying common risk factors for complications and readmission.

Materials and methods: A cross-sectional study was conducted at King Abdullah Specialist Children’s Hospital (KASCH), Riyadh, Kingdom of Saudi Arabia, using the BESTCare 2.0 Health Information System (SKHIC, Riyadh, Saudi Arabia). All cases admitted under the pediatric ambulatory surgery unit from June 2015 to May 2018 were included. We reviewed 462 medical charts and recorded the variables of age, sex, American Society of Anesthesiologists (ASA) classification, complications, and readmission within one month of the surgery. SPSS (IBM Corp., Armonk, NY, USA) was used for data analysis. Associations between exposure variables (e.g., age, duration of surgery) and the outcome variables (e.g., rate of readmission and complications) were measured using the Chi-square test for categorical variables, the T-test and analysis of variance (ANOVA) for numerical and categorical variables, and logistic regression for multiple variables to control confounding variables.

Results: Approximately, 3.5% of the pediatric ambulatory surgery cases required readmission, and 10.6% of the patients had complications with zero mortality. All the variables had no significant association either with the readmission or complications (p > 0.05), except for the duration of surgery in minutes which was associated with complications (OR 1.006, 95% CI, 1.000-1.012, P = 0.035).

Conclusion: Among pediatric ambulatory surgery cases, the mortality rate is 0%, with low complications and readmission rates. Also, the longer the surgery, the higher the risk of complications.

## Introduction

Ambulatory surgery can be described as surgery that is performed on outpatients who are discharged on the same day [1]. Ambulatory surgery has several advantages, including lower costs, increased provider productivity, and increased patient convenience [2]. In North America, two-thirds of the surgical trials are completed in ambulatory settings [3]. Ambulatory surgery cases have been rapidly increasing because of their low percentage of complications, quality in various aspects, and lower cost, approaching 30% of Medicare surgical expenses in 2014 [4,5]. There had been a few readmission cases to the hospital for several reasons, and most of them did not have major complications [1]. A readmission is defined as a subsequent inpatient admission to an acute care facility occurring within 30 days of the discharge date of an eligible index admission [6].

Globally, a study done in the United States showed that the overall readmission rate was 7.6% after pediatric tonsillectomy [7]. Among readmissions, 6.3% went to the ambulatory surgery center, 77.5% revisited the ER, and 16.2% were readmitted as inpatients [7]. Also, at the ambulatory surgery center of Toronto Western Hospital, 1.1% returned to the hospital for readmission within 30 days. Of all cases, 3% of the readmissions returned to the ER, 92% were readmitted to the ambulatory surgery center, and 5% were readmitted as inpatients [8]. 

To the best of our knowledge, the safety, readmission, and complications of pediatric ambulatory surgery in our population have not been studied. Therefore, this research aimed to assess the safety of pediatric ambulatory surgery, determine the prevalence of readmission and complications, and evaluate the outcome of ambulatory surgery.

## Materials and methods

This was a single-centered, cross-sectional study that was conducted at King Abdullah Specialist Children’s Hospital (KASCH). KASCH is a tertiary care center that is located in Riyadh, Kingdom of Saudi Arabia, with a capacity of 600 beds. The BESTCare 2.0 Health Information System (SKHIC, Riyadh, Saudi Arabia) was used for data collection. Charts of pediatric patients admitted to the ambulatory surgery unit from June 2015 to May 2018 were reviewed to assess the safety and rate of readmission. The study included all pediatric ambulatory surgery cases in both male and female patients from all nationalities up to the age of 14 years (which is the designated age group for pediatrics in Saudi Arabia) and the American Society of Anesthesiologists (ASA) class I-II. All planned readmission cases were excluded from the study.

Outcome, complications, and readmission were compared among the included variables. The included variables were age, sex, nationality, weight, height, ASA class, type of anesthesia, and duration of surgery. For the sample size, the OpenEpi Version 3.01 sample calculator was used with a margin of error of 5% and a confidence level of 95%. With a prevalence rate of 7.6% that was obtained from previous research [7], the sample size was 462 cases. The sampling technique that was used to represent the population was random sampling from all ambulatory surgery cases by using Microsoft Excel 2016.

For data analysis, the Statistical Package for Social Sciences (SPSS), version 20 (IBM Corp., Armonk, NY, USA) was used. A set of statistical tests was used to assess the association and significance between the exposure variables (e.g., age, ASA class, sex, duration of surgery, type of anesthesia) and the outcome variable (e.g., rate of readmission and complications). The T-test and analysis of variance (ANOVA) were used to measure the association between numerical and categorical variables (e.g., comparing the means) and Chi-square for categorical variables (e.g., sex relation to readmission). The categorical variables were presented as frequencies and percentages, while the numerical variables were presented as mean and standard deviation. To study the effect of multiple variables (both categorical and numerical), logistic regression was used, and the odds ratio was used to test the outcome effect on the variables. A test was considered significant if the p-value was less than 0.05.

For ethical considerations, since this research is a chart review, there was no need for consent. Data collection was done after having approval from the King Abdullah International Medical Research Center (KAIMRC). During the study, security procedures (e.g., encryption, password protection) were followed when patient data were transferred into SPSS on the computer. Any information that identified the patient was removed and replaced with a code.

## Results

Out of 462 patients, male patients (n = 303, 65.6%) undergoing ambulatory surgery were more than females (n = 159, 34.3%). More than half of the patients were ASA I (n = 291, 63%), and the largest age group was school age (6-12 years; n = 146, 31.6%; Table 1).

**Table 1:** Variables ASA: American Society of Anesthesiologists.

	Number n = 462
Sex	
Male	303 (65.6%)
Female	159 (34.4%)
ASA	
I	291 (63%)
II	171 (37%)
Age group	
0–12 months)	56 (12.1%)
1–3 years	109 (23.6%)
3–6 years	124 (26.8%)
6–12 years	146 (31.6%)
12–14 years	27 (5.8%)

Complications and readmission rates

As shown in Table 2, the complication rate for patients who had ambulatory surgery was low (n = 49, 10.6%). Also, the readmission rate was low (n = 16, 3.5%), and no mortality was identified.

**Table 2:** Complications and readmission rates

	N = 462
Complications	
Yes	49 (10.6%)
No	413 (89.4%)
Readmission	
Yes	16 (3.5%)
No	446 (96.5%)
	No mortality was identified

Association between the variables, readmission, and complications

In Table 3, Chi-square tests of independence were calculated to compare the frequency of readmission in the categorical variables (sex, ASA). No significant association was found in both sexes (X2 [1, N = 462] = 3.501, p = 0.061) and ASA (X2 [1, N = 462] = 1.199, p = 0.274). Also, independent-samples t-tests were conducted to assess the association between each numerical variable (age, duration of surgery) and the readmission. There was no significant difference in age between patients who were readmitted (M = 3.99, SD = 3.37) and those who were not readmitted (M = 4.76, SD = 3.6); t (460) = 0.85, p = 0.398. For the duration of the surgery, there was no significant difference between readmitted patients (M = 44.56, SD = 40.44) and those who were not readmitted (M = 46.93, SD = 40.69); t (460) = 0.229, p = 0.819. These results suggest that age and duration of surgery do not affect readmission.

**Table 3:** Association between the variables and readmission ASA: American Society of Anesthesiologists, M: mean, SD: standard deviation.

	No n = 446		Yes n = 16		P-value
Sex					
Male	296 (66.4%)		7 (43.8%)		0.061
Female	150 (33.6%)		9 (56.3%)		
ASA					
I	283 (63.5%)		8 (50%)		0.274
II	163 (36.5%)		8 (50%)		
	M	SD	M	SD	
Age (years)	4.76	3.37	0.398	3.37	0.398
Duration of surgery (minutes)	46.93	40.44	0.819	40.44	0.819

The same tests used for the readmission were used with the complications with the same variables in Table 4. No significant association was found in both sexes (X2 [1, N = 462] = 1.731, p = 0.188) and ASA (X2 [1, N = 462] = 2.316, p = 0.128). Also, there was no significant difference in age between patients who had complications (M = 4.9, SD = 3.597) and those who had no complications (M = 4.72, SD = 3.597); t (460) = −0.334, p = 0739. For the duration of the surgery, there was a significant difference between patients with complications (M = 58.82, SD = 62.11) and those who had no complications (M = 45.43, SD = 37.14); t (460) = −0.33, p = 0.029. These results suggest that the only variable that had an association with complications was the duration of surgery.

**Table 4:** Association between the variables and complications ASA: American Society of Anesthesiologists, M: mean, SD: standard deviation.

	No n = 413		Yes n = 49		P-value
Sex					
Male	275 (66.6%)		28 (57.1%)		1.88
Female	138 (33.4%)		21 (42.9%)		
ASA					
I	265 (64.2%)		26 (53.1%)		0.128
II	148 (35.9)		8 (46.9%)		
	M	SD	M	SD	
Age (years)	4.72	3.597	0.739	3.597	0.739
Duration of surgery (minutes)	45.43	62.11	0.029	62.11	0.029

To find any confounding variables, multivariate analysis for complication covariant was used as in Table 5. Duration of surgery had a significant association with having complications (odds ratio [OR] of 1.006, 95% confidence interval [CI], 1.000-1.012, P = 0.035).

**Table 5:** Multivariate analysis for complication covariant ASA: American Society of Anesthesiologists, OR: odds ratio, CI: confidence interval.

	OR	95% CI	P-value
Age	1.008	0.927–1.095	0.856
Duration of surgery, min	1.006	1.000–1.012	0.035
ASA			
I	Reference		
II	0.644	0.353–1.176	0.152
Sex			
Male	Reference		
Female	0.646	0.351–1.189	0.160

## Discussion

The popularity of ambulatory surgery is rising because of its cost-saving and convenience [1,2]. The low rate of adverse events or complications during the intraoperative or immediate postoperative periods further justifies the rapid growth of ambulatory surgery and its potential to be the standard of care [4,5]. 

To the best of our knowledge, this article is the first to assess the rates of readmission and complications in pediatric ambulatory surgery in a tertiary hospital. In addition to cost-effectiveness and convenience, our data show that pediatric ambulatory surgery is safe with a 0% mortality rate, a low readmission rate of 3.5%, and a complication rate of 10.6% that happened within 30 days post-ambulatory surgery. These rates are in the same range as in previously published studies. 

Within two weeks after their ambulatory surgery [3], Natof's study reported 106 major complications among 13,433 ambulatory surgical patients, a complication rate of 0.79%. Warner et al. found that 33 of 38,598 patients undergoing ambulatory surgery (i.e., 0.09% of the patients) had major complications or died within 1 month after their ambulatory procedures, which is considered insignificant [9]. In another study, Heino et al. included 500 ambulatory surgeries, and 41 of them visited a doctor within one month of their surgery. The main reasons for this visit were pain in 100% of them, minor bleeding in 58.5% of them, and inflammation in 39.0% of the patients. None of the above studies, however, reported what percentage of patients needed hospital admission [10]. Henderson et al. identified emergency readmission rates of up to 2.3% within 28 days after ambulatory surgery, and the readmission cases were dependent on the type of surgery [11]. Twersky et al. found that out of 6243 ambulatory surgical patients, only 187 (3%) had return visits within 30 days of their surgery; 44% of those visits resulted from surgical complications [12]. 

Differences between the studies could be attributed to various factors, such as differences in the quality of surgical care, differences in the surgeons’ skills and clinical judgment, or significant differences in the patient populations and the types of completed surgical procedures. The absence of mortality in our study was consistent with the above-mentioned articles. Only Warner et al., who had the largest patient population, reported deaths, and even their reported death rate was very low (4 in 38,598) [9].

The readmission rate is 0.7% to 2.3% in ambulatory care surgeries, and our study’s rate is 3.5% [11,13]. Also, similar studies showed that the complication rates range from 0.05% to 20% in ambulatory care surgeries, and the study’s complication rate is compatible [14]. Regarding the complications, an overestimation of them may have occurred. Every emergency room visit with a complication that had minimal relation to the surgery within one month was documented to make sure that there were no complications missed, and the complication rate was still within the worldwide readmission rate range. 

Regarding the variables (age, sex, ASA), they were not associated with neither the complications nor the readmission. The only variable that showed an association with the complications was the duration of surgery in minutes, as for every 10 minutes there is a 6% higher risk of having complications. 

This study was done in only one center (KASCH), and this is one of the limitations. Also, this study is a chart review using BESTCare 2.0 Health Information System in one center, so any patient visits to other facilities with a complication or required readmission would not be reported.

## Conclusions

This research showed that pediatric ambulatory surgery is safe, with 0% mortality and low complication and readmission rates. We can conclude that this type of surgery is a good alternative to inpatient-based care. With a longer duration of surgery, there will be a higher risk of complications. We recommend national health care facilities to invest more in pediatric ambulatory surgery. However, due to the limitations of this research, we encourage a national multicentric study to evaluate the safety, convenience, and cost-effectiveness of pediatric ambulatory surgery.
